# Tracking hantavirus nucleocapsid protein using intracellular antibodies

**DOI:** 10.1186/1743-422X-7-339

**Published:** 2010-11-24

**Authors:** Jiandong Li, Quanfu Zhang, Tao Wang, Chuan Li, Mifang Liang, Dexin Li

**Affiliations:** 1 State Key Laboratory for Infectious Disease Control and Prevention, Institute for Viral Disease Control and Prevention, China CDC, 155 Changbai Road, Changping District, Beijing 102206, PR China; 2 State Key Laboratory Molecular Virology and Genetic Engineering, Institute for Viral Disease Control and Prevention, China CDC, 155 Changbai Road, Changping District, Beijing 102206, PR China

## Abstract

**Background:**

Hantavirus nucleocapsid (N) protein is a multifunctional viral macromolecule involved in multiple stages of the viral replication cycle. The intracellular trafficking of N protein during virus assembly remains unclear.

**Methods:**

We used N protein-specific intracellular expressed antibodies to track the localization and distribution of Hantaan virus and Seoul virus N protein. The N protein-specific antibody single-chain variable antibody fragments (scFvs), which bind an N-terminal linear epitope (L13F3) and C-terminal conformational domain (H34), were intracellularly expressed in the endoplasmic reticulum (ER) by fusion of the SEKDEL retention signal peptide at the carboxyl terminus, and in the cytoplasm (Cyto) by deletion of the ER membrane target signal peptide. Stable Vero-E6 cell lines expressing intracellular scFvs were either infected with hantavirus or transfected with an N protein expression plasmid; virus replication and N protein intracellular localization were determined.

**Result:**

N protein co-localized with scFvs in the ER and cytoplasm with or without viral membrane glycoproteins. Hantavirus replication was inhibited in both the scFvs-ER- and scFvs-Cyto-expressing stable cell lines.

**Conclusion:**

N protein may be expressed in the ER retention signal peptide of KDEL circulating region (ER/*cis*-Golgi) without the assistance of G protein, and so expression of N protein in both the cytoplasm and within the ER/*cis*-Golgi plays an important role in virus replication.

## 1. Background

Hantaviruses are members of the Bunyaviridae family, which contain three negative-sense, single-stranded RNA genome segments designated large (L), medium (M), and small (S) [1]. The S, M, and L segments encode the nucleocapsid protein (N), glycoproteins (Gn and Gc), and L protein (an RNA-dependent RNA polymerase), respectively. Hantaviruses do not have matrix proteins, but the N protein has been proposed to play a key role in virus assembly [2]. N protein is expressed in the cytoplasm, viral glycoproteins are co-translated in the endoplasmic reticulum (ER), once cleaved, Gn and Gc undergo glycosylation, folding, and heterodimerization in the Golgi complex, where they are retained and accumulate. For assembly to occur, N as well as Gn and Gc, must move to the same intracellular location. After interaction of N protein with viral RNA and subsequent assembly, ribonucleoprotein (RNP) is targeted to the Golgi complex by specific recognition of the cytoplasmic tail of Gn and Gc protein [3], the interaction of Gn protein cytoplasmic tail and the middle domain of the N protein was suggested to play essential role to direct RNPs to the site of the virus assembly [4] and the complete hetero-oligomeric (Gn-Gc) spike complex of hantaviruses might mediates the packaging of RNP into virions [5].

N protein has an intrinsic RNA chaperone activity, which is important for encapsidation and genome replication [6,7]. The RNA-binding domain of N protein is situated within a central conserved region between residues 175 and 217 [8]. The 141 residues proximal to the C-terminal are required for Golgi localization [9]. Both N- and C-terminal regions have been implicated in homotypic N protein interaction, and putative coiled-coil motifs in the N-terminal region of N protein have been proposed to facilitate trimerization [10-12]. N was not observed in the Golgi so far, but it could be observed to surround the Golgi after infection [13,9] and it was shown that targeting of N protein to the ER/Golgi intermediate compartment (ERGIC), prior to its movement to the Golgi compartment, and an intact ERGIC are necessary for viral replication [14]. However, the impact of N protein intracellular trafficking on the cell and its effect on virus replication remain unclear. We used intracellular expression of anti-Hantaan virus (HTNV) and Seoul virus (SEOV) N protein N-terminal- and C-terminal-specific antibodies, respectively, to block or knock down N protein function at targeted sites, with or without co-expressed membrane glycoproteins, and assess the effect on virus replication and N protein intracellular trafficking. Our data showed that N protein co-localized with both cytoplasm and ER-retarded antibodies either with or without the help of G protein and virus replication was inhibited by related intracellular antibodies. These data suggest, therefore, that presentation of N protein both in the cytoplasm and within the ER/*cis*-Golgi plays an important role in hantavirus replication.

## 2. Materials and methods

### 2.1. Cells and antibodies

Vero-E6 cells, COS-7 cells, and a mouse hybridoma cell line L13F3 expressing mouse mAb binding to N protein of HTNV and SEOV (which targeted at a N-terminus epitope [15]) were cultured in Dulbecco's modified Eagle's medium (DMEM) supplemented with fetal calf serum (FCS; 10% v/v), penicillin (100 IU/ml), streptomycin (100 μg/ml), and L-glutamine complete (4.5 mM). The phage display-derived human F_ab _H34, which recognizes the HTNV N protein C-terminus conformational domain, was produced in our laboratory [16].

### 2.2. Plasmids

To construct single-chain fragment variable antibody fragments (ScFvs) specific for hantaviruses N protein, mRNA was isolated from ~1 × 10^6 ^L13F3 hybridoma cells using Trizol reagent (Invitrogen, Carlsbad, CA, USA). The first cDNA strand was synthesized using SuperScript™reverse transcriptase (Invitrogen) according to the manufacturer's directions. L13F3 heavy chain (VH) variable region gene fragments were amplified using the forward primer 5'-GAATAGGCCATGGCGGAGGTCCAGCTGCAGGAGTCTGGGGGAGGCTTA G-3' and the reverse primer 5'-GGCCAGTGGATAAAGCTTTGGGGGTGTCGTTTTGGC-3'. *Nco*I and *Hin*dIII restriction sites were introduced at the 5'- and 3'-ends, respectively, of the VH gene using synthetic oligonucleotides. The L13F3 Vκ gene was PCR-amplified using a forward primer with the nucleotide sequence 5'-TACAGGATCCACGCGTAGACATTGTGATGACCCAGTCT-3' and a reverse primer of sequence 5'-TGACAAGCTTGCGGCCGCGGATACAGTTGGTGCAGCATC-3', containing MluI and NotI sites, respectively. PCR products were subsequently inserted into plasmid pOPE-101-215 [17] between the restriction sites. The resulting plasmid was named pOPE-L13F3-scFv. As above, the H34-scFv-expressing plasmid pOPE-H34-scFv was constructed as follows: the H34 VH gene was amplified from the H34 F_ab _fragment using synthetic oligonucleotides with sequences 5'-GAATAGGCCATGGCGGAGGTGCAGCTGGAGTCT-3' and 5'-CAGTCAAGCTTTGATGAGACGGATACC-3', and *Nco*I and *Hin*dIII restriction sites were introduced at the 5'- and 3'-termini, respectively. The H34 V_K _gene was amplified using a forward primer with the nucleotide sequence 5'-TACAGGATCCACGCGTAGAAATTGTGTTGACGCAGTCTCCA-3' and the reverse primer 5'-TGACAAGCTTGCGGCCGCGAAGACAGATGGTGCAGCCACAGTTCGTCTG A-3', and MluI and NotI restriction sites were introduced at the 5'- and 3'-ends, respectively. PCR products were cloned into the plasmid pOPE-101-215.

To construct plasmids expressing intracellular scFvs in mammalian cells, the scFv gene (encoding a 6-histidine tag at the 3'-terminus) was PCR-amplified using the forward primers scFv-L13F3F-BssHII 5'-GGCGCGCACTCCGAGGTCCAGCTGCAGGAGTCTG-3', scFv-H34F-BssHII 5'-GGCGCGCACTCCGAGGTGCAGCTGGAGTCT-3' and the reverse primer scFv-R-XhoI 5'-CTCGAGATGATGATGGTGATGATGGGATAG-3' from plasmids pOPE-L13F3-scFv and pOPE-H34-scFv, respectively. Amplified fragments were cloned into pEF/myc/ER (Invitrogen) between the BssHII and XhoI restriction sites, producing the intracellular antibody expressing plasmids L13F3-scFv-ER and H34-scFv-ER. For the construction of plasmids L13F3-scFv-CYTO and H34-scFv-CYTO expressing cytoplasmic scFv, the scFv gene was prepared using NcoI/NotI from plasmids pOPE-L13F3-scFv and pOPE-H34-scFv and inserted into the pEF/myc/cyto vector (Invitrogen) between the *Nco*I and *Not*I restriction sites.

Plasmid pEF-N76118 was generated by amplifying the HTNV strain 76-118 N protein coding sequence using primers carrying adapters, and then cloned into pEF/myc/cyto and digestion with *Nco*I and *Xba*I.

### 2.3. Transfection

The HTNV 76-118 N protein mammalian expression plasmid pEF-N76118 and intracellular antibody-expressing plasmids L13F3-scFv-ER, H34-scFv-ER, L13F3-scFv-CYTO, and H34-scFv-CYTO were transiently or stably transfected into eukaryotic cells. Plasmid DNA (3 μg or 1 μg) was coated onto the bottom of each well of a 6- or 24-well plate, respectively, using Lipofectamine 2000 (Invitrogen) according to the manufacturer's instructions. COS-7 cells were co-transfected with pEF-N76118 and one of the antibody-expressing plasmids. Cells were harvested 48 h after transfection. To generate cell lines for stable expression of antibodies, Vero E6 cells were transfected with scFv-expressing plasmids; cultures supplemented with G418 (500 μg/ml) were started 48 h after transfection. Mock-transfected cells died within 7 days in selective medium. Clones were isolated using the limited dilution method, dispensed into 96-well plates, and kept under selection pressure (G418; 500 μg/ml) for 14 days before reverting to normal culture medium. In this way, the antibody-expressing, stable Vero-E6 cell lines L13F3-scFv-ER-E6, H34-scFv-ER-E6, L13F3-scFv-CYTO-E6, and H34-scFv-CYTO-E6 were obtained.

### 2.4. Immunoblot assay

Immunoblots were used to confirm specificity of the recombinant scFvs. Routinely concentrated HTNV strain 76-118 and SEOV strain L99 prepared from Vero E6 cells (8 μl) were mixed with 5× SDS-PAGE Laemmli sample buffer [2 μl; 60 mM Tris-HCl (pH 6.8), 2% (w/v) SDS, 10% (v/v) glycerol, 5% (v/v) β-mercaptoethanol, and 0.01% (w/v) bromophenol blue]. Following denaturing at 95°C for 10 min, proteins were resolved on 12% (w/v) SDS-PAGE gels and electroblotted onto PVDF membranes. These membranes were rinsed with phosphate-buffered saline (PBS), blocked using 5% (w/v) skim milk powder in PBS (PBS-M) for 30 min, and probed with recombinant L13F3-scFv and H34-scFv (10 ml; 1 μg/ml) from XL1-Blue in PBS-M at room temperature (RT) for 2 h. After washing, the membrane was incubated in mouse anti-penta-His monoclonal antibody (1:1000 in PBS-M) for 1 h at RT. Membranes were then washed and incubated with goat anti-mouse IgG gamma chain-specific AP-conjugated antibody (1:1000 dilution in PBS-M) (Sigma) for 1 h at RT. After washing, membranes were developed in BCIP/NBT (Roth, Karlsruhe, Germany) substrate solution. All washes were performed thrice for 5 min each in 0.05% (v/v) PBS-T.

### 2.5. Enzyme-linked immunosorbent assay (ELISA)

To determine the concentration of viral particles in culture supernatants, group-specific L13F3 mouse anti-N mAb was coated onto 96-well microtiter plates overnight at 4°C. Coated plates were blocked with 5% (w/v) skimmed milk in PBS, and serial dilutions of detergent-treated culture supernatants were added. After washing, bound viral particles were detected by horseradish peroxidase-conjugated L13F3 mAb diluted 1:1000 in PBS-T. After washing three times with PBS-T, TMB peroxidase substrate [100 μl; dimethylformamide and hydrogen peroxide (H_2_O_2_)] was added and developed at RT for ~30 min. The reaction was stopped by adding H_2_SO_4 _(100 μl; 1 N). A_450 _(reference 620 nm) was read using a DTX 880 multi-mode detector (Beckman Coulter, Fullerton, CA, USA). The cutoff value was three standard deviations above the mean absorbance of the negative control wells.

### 2.6. Virus infection

HTNV strain 76-118 and SEOV strain L99 were propagated on Vero E6 cells. Cell cultures were harvested at 10 days postinfection; cell debris was removed by centrifugation at 4000 × *g *and samples stored in 1-ml aliquots at -80°C. Vero E6 cells and the intracellular antibody-expressing Vero-E6 cell lines L13F3-scFv-ER-E6, H34-scFv-ER-E6, L13F3-scFv-CYTO-E6, and H34-scFv-CYTO-E6 were cultured in T25 flasks to 85% confluence; cells were washed twice with pre-warmed serum-free DMEM and virus 76-118 and L99 were loaded at a multiplicity of infection (MOI) level of 0.01. Cells were then incubated at 37°C for 2 h. Cells were washed twice with pre-warmed serum-free DMEM and maintained in DMEM (5 ml) supplemented with 2% (v/v) FBS and incubated at 37°C for 8 days. Cell culture supernatants (1.0 ml) were collected each day after the third day postinfection and an identical amount of fresh medium was added. Viral antigen levels were assayed by ELISA using the L13F3 mAb, as described above. Supernatant Hantaviruses titers were determined by serially diluting supernatants of each virus stock and performing quadruplicate infections on Vero-E6 cells in 96-well plates. The TCID50 assay endpoint was determined on day 10. Used medium was discarded and cells fixed in 80% (v/v) ice-cold acetone. Viral antigen were detected by horseradish peroxidase conjugated L13F3 monoclonal antibody, TMB peroxidase substrate (Dimethylformamide and hydrogen peroxide) was added to develop color. Absorbance at 450 nm (reference 620 nm) was read with a DTX 880 multi-mode detector (Beckman Coulter). The cut-off value was determined by the 3 times SD above the mean absorbance of the negative control wells. And these were addressed at the part of methods. The TCID50 value was calculated by the method of Reed and Muench [18].

### 2.7. Immunofluorescence assay (IFA)

Specificity of the scFvs and intracellular distributions of N protein and intracellular scFvs were determined by indirect immunofluorescence assay (IFA). Hantaviruses-infected Vero E6 cells, stably transfected Vero E6 cells, or transiently transfected COS-7 cells were passaged onto sterile coverslips in six-well plates overnight. Cells were fixed by treatment with methanol for 10 min at -20°C followed by acetone for 10 min at -20°C. To eliminate nonspecific binding, cells were pre-incubated with PBS containing 5% (v/v) FCS for 30 min at RT in a humidity chamber. Antibody dilutions of 1:50 for H34 IgG, 1:100 for L13F3 mAb, 5 μg/ml of purified anti-N protein scFvs, and 1:200 for anti-penta-His mAb in PBS containing 5% FCS were used. The first round of incubation was carried out for 1 h at RT. For detection of bound, purified anti-N protein scFvs, the secondary antibody (mouse anti-His tag mAb) was loaded and incubated for 1 h. Antibodies were detected using either an antihuman fluorescein isothiocyanate (FITC)-conjugated secondary antibody or anti-mouse tetramethyl rhodamine isothiocyanate (TRITC)-conjugated antibody. Coverslips were washed in PBS, mounted on glass slides, and imaged under a TCS NT confocal microscope (Leica, Wetzlar, Germany).

### 2.8. His tag pull-down assay

About 5 × 10^7 ^HTNV-infected Vero E6 cells or the stably transfected cell lines L13F3-scFv-ER-E6, H34-scFv-ER-E6, L13F3-scFv-CYTO-E6, and H34-scFv-CYTO-E6 were harvested by centrifugation. Pellets were lysed in 1 ml ice-cold extraction buffer [TBS, pH 7.5; containing phenylmethylsulfonyl fluoride (1 mM), aprotinin (5 μg/ml), leupeptin (5 μg/ml), and 1% (v/v) Triton X-100] and gently rotated for 30 min. Samples were centrifuged at 4°C and 12,000 rpm for 10 min to remove cell debris. Ni-NTA His-bind resin (200 μl; Stratagene, La Jolla, CA, USA) was added and cleared supernatants were incubated at 4°C and 600 rpm in an Eppendorf thermomixer for 2 h. The resin was then harvested by centrifugation for 2 min at 4000 × *g *at 4°C and washed five times with 1 ml PBS and then five times with 1 ml PBS containing imidazole (20 mM). Bound proteins were eluted from the resin by incubation at 4°C in 500 μl PBS containing 500 mM imidazole. Pull-down elutes were stored at -80°C in 100-μl aliquots. Aliquots were concentrated by adding two volumes of ice-cold 100% ethanol and centrifuged at 10,000 × *g *for 10 min; pellets were resuspended in 10 μl dH_2_O followed by analysis by SDS-PAGE and immunoblotting with anti-penta-HIS (Qiagen, Hilden, Germany) and L13F3 N protein-specific mAbs as described above.

## 3. Results

### 3.1. Intracellular expression of L13F3, H34 scFvs

**Figure 1:**
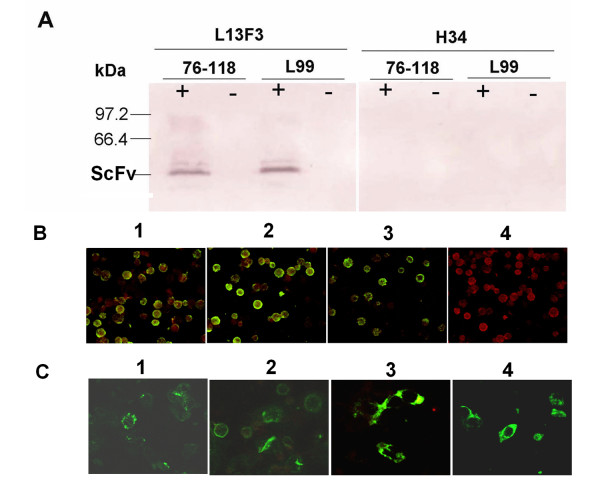
**Characterization of recombinant scFv antibodies**. A) Immunoblot analysis of recombinant scFvs. Concentrated 76-118 and L99 virus were separated on 12% SDS-PAGE and blotted on PVDF membrane, parallel treated normal vero E6 cells culture supernatant was used as negative control, the membrane was incubated with prokaryotic produced and purified scFv, and mouse anti- penta-His tag monoclonal antibody, detected with horseradish peroxidase-conjugated goat anti-mouse antibody. B) Immunofluorescence analysis of recombinant scFvs, recombinant N protein expressing SF9 cells were fixed on dot slides, incubated with scFvs, (1) 76-118 N incubate with L13F scFv; (2) L99 N incubate with L13F scFv; (3) 76-118 N incubate with H34 scFv; (4) L99 N incubate with H34 scFv. C) Intracellular localization of scFvs. COS-7 cells were seeded onto a 6-well plate, grown to 80% confluency, and transfected with intracellular antibodies expressing plasmid (1) L13F3-scFv-CYTO; (2) H34-scFv-CYTO; (3) L13F3-scFv-ER and (4) H34-scFv-ER. The cells were fixed at 48 h posttransfection, and the scFvs was identified with IFA by using anti-His MAb, images were obtained with confocal microscope with 100 × objectives.

To determine the binding activity of the recombinant scFvs, immunoblot and IFA and ELISA analyses on HTNV strain 76-118 and SEOV strain L99 were performed. Figure 1 depicts an immunoblot using L13F3 (Figure 1A, left panel) and H34 scFvs (Figure 1A, right panel) to probe viruses. The L13F3 scFv showed binding to both strain 76-118 and L99; this finding was corroborated by IFA data (Figure 1B, panel 1 and 2). The H34 scFvs bound only to HTNV strain 76-118 (Figure 1B, panel 3) and not SEOV stain L99 (Figure 1B, panel 4) in the IFA test, and no binding was detected by immunoblotting (Figure 1A, right panel). These data are consistent with the binding activities of the L13F3 and H34 parent antibodies.

**Figure 2:**
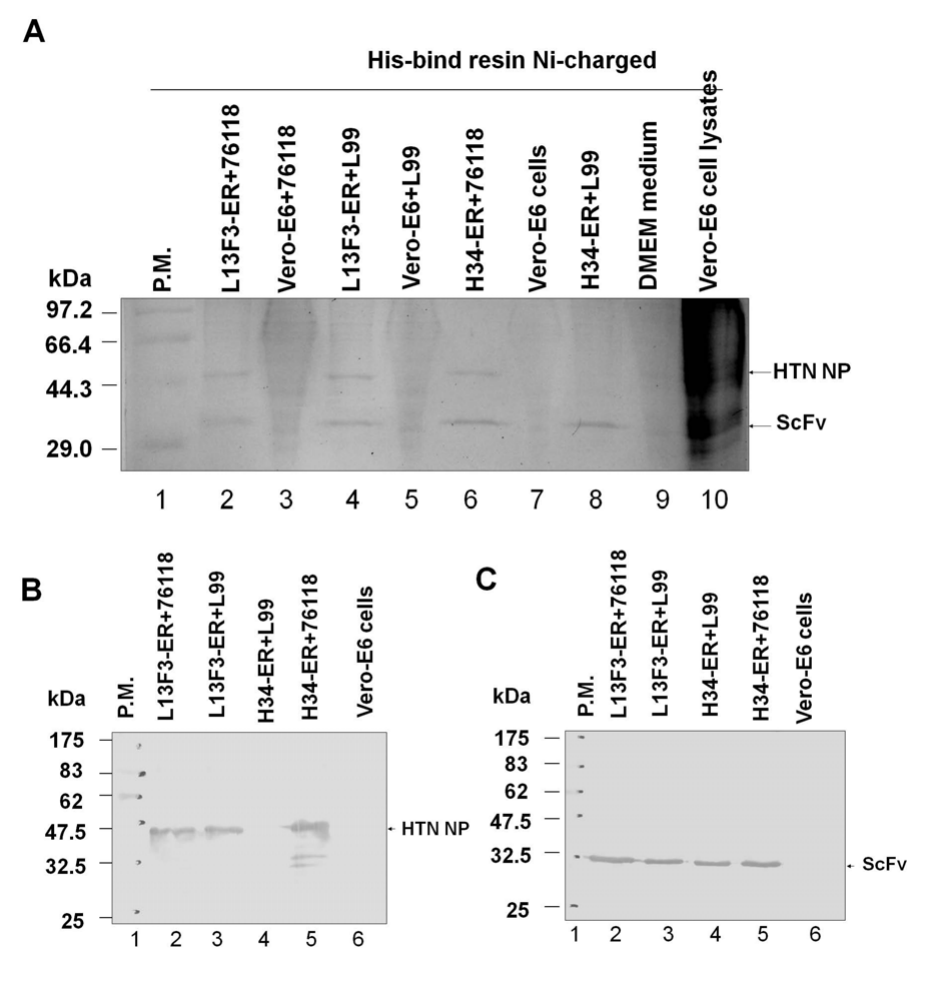
**His-tag pull-down assay between intracellular scFvs and hantavirus N protein. Stably transfected Vero E6 cells, indicated as L13F3-scFv-ER and H34-scFv-ER, were infected with hantavirus 76-118 and L99 respectively**. About 5 × 10^7 ^infected cells were collected and cell extracts incubated with Ni-NTA His-bind resin. After washing, bound proteins were eluted and concentrated by adding two volumes of ice-cold 100% ethanol and centrifuged at 10,000 × *g *for 10 min. Pellets were resuspended in 10 μl distilled water and analyzed by SDS-PAGE (A), Intracellular scFvs were analysized by immunoblotting with anti-penta-HIS (Qiagen) (B), and N proteins were blotted by N protein-specific mAbs L13F3 (C).

**Figure 3:**
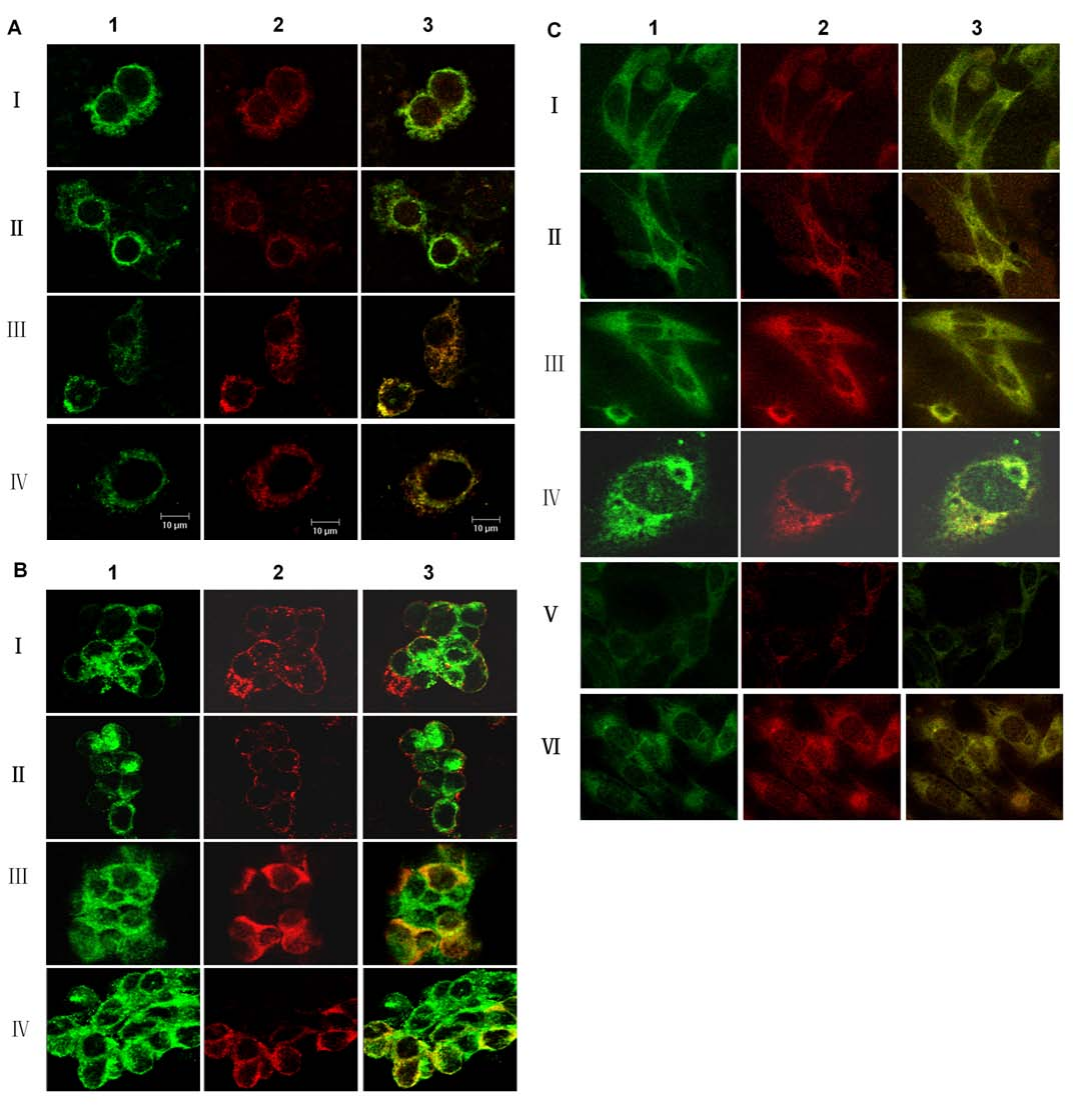
**Co-localization of HTNV N protein with intracellular scFvs**. A) Co-localization of recombinant N protein with intracellular scFvs, COS-7 cells were co-transfected with pEF-N76118 and a intracellular scFv expressing plasmid [(I) L13F3 scFv-CYTO, (II) H34-scFv-CYTO, (III) L13F3-scFv-ER, (IV) H34-scFv-ER], 2 days post transfection, slides were acetone fixed, and costained with human anti-N IgG H34 [green, panels 1] or anti-penda-His mAb [red, panels 2]), merged images of the panels 1 and 2 are presented in panels 3; B) Co-localization of Gc with intracellular scFvs, Stably transfected vero E6 cells [(I) L13F3 scFv-CYTO, (II) H34-scFv-CYTO, (III) L13F3-scFv-ER, (IV) H34-scFv-ER] were infected with 76-118 at an MOI of 0.1, and after 6 days slides were acetone fixed, and costained with anti-Gc mAb AH100 [green, panels 1] or anti-N mAb [red, panels 2]), merged images of the panels 1 and 2 are presented in panels 3. C) Co-localization of viral N protein with intracellular scFvs. Intracellular scFv stably transfected vero E6 cells were infected with 76-118 at an MOI of 0.01, [(I) L13F3 scFv-CYTO, (II) H34-scFv-CYTO, (III) L13F3-scFv-ER, (IV) H34-scFv-ER], or with L99 at an MOI of 0.01 [(V) L13F3 scFv-CYTO, (VI) L13F3-scFv-ER], and after 5-6 days slides were acetone fixed, and costained with human anti-N mAb AH34 [green, panels 1] or mouse anti-His tag mAb [red, panels 2]), merged images of the panels 1 and 2 are presented in panels 3. Excitation wavelengths were 488 and 568 nm; Fluorescence images were obtained with confocal microscope using 100 × objectives

**Figure 4:**
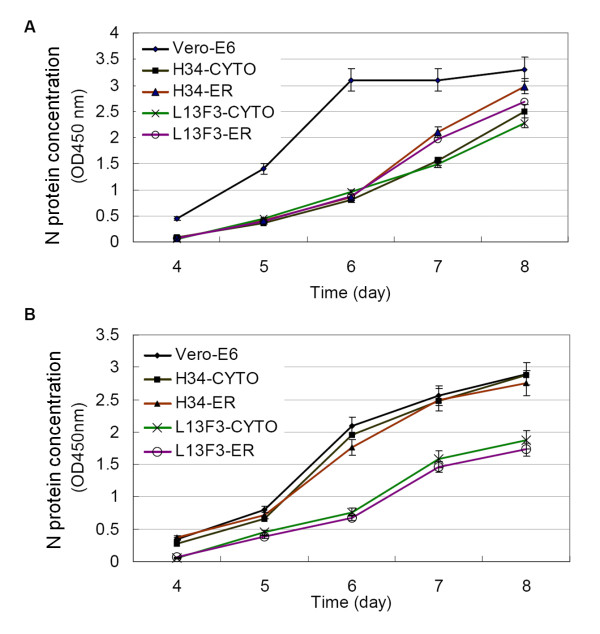
**Hantavirus N protein levels in supernatants of virus infected cell lines determined by ELISA**. (A) Infected with HTNV strain 76-118 and (B) infected with SEOV strain L99. Mouse anti-N mAb L13F3 was coated onto 96-well microtiter plates; detergent-treated culture supernatants were loaded and N protein was detected by horseradish peroxidase-conjugated anti-HTNV L13F3 mAb. Error bars represent the standard deviation of three independent experiments.

Intracellular localization of scFvs was determined by indirect immunofluorescence. As expected, L13F3 and H34 scFvs-Cyto accumulated in the cytoplasm of transfected cells (Figure 1C, 1 and 2), while L13F3 and H34 scFvs-ER yield a typical ER specific morphological fluorescence image (Figure 1C, 3 and 4).

### 3.2. N protein interacted with intracellular antibodies in vivo

To investigate whether N protein interacts with intracellular scFvs in vivo, a His-tag pull-down experiment was performed. To exclude any nonspecific binding to His-bind resin, 76-118 or L99 virus-infected Vero-E6 cells were used as negative controls and normal Vero-E6 cells as blank controls. N protein and intracellular scFvs were detected by SDS-PAGE analysis of the pull-down eluates (Figure 2A, lanes 2, 4, and 6). However, scFv H34, which proved to be a HTNV type-specific antibody, did not pull-down the N protein of Seoul L99 virus, as expected (Figure 2A, lane 8). N protein and scFvs were not found in either negative or blank controls (Figure 2A, lanes 3 and 5). These observations were confirmed by detection with either anti-N protein mAb (Figure 2B) or anti-His mAb (Figure 2C) in immunoblot assays. Therefore, N protein and the N protein-specific scFvs likely specifically interact in vivo.

### 3.3. Hantavirus N protein co-localized with intracellular antibodies

Intracellular localization of N protein in transiently co-transfected COS-7 cells or virus infected Vero-E6 cells containing intracellular antibodies was determined under a confocal microscope. The recombinant N protein of HTNV strain 76-118 co-localized with intracellular L13F3 and H34 scFvs in both the ER and cytoplasm in COS-7 cells (Figure 3A). In contrast, the cytoplasmically expressed scFv did not co-localize with Gc protein of HTNV strain 76-118, but the ER-specific scFvs showed partial co-localization (Figure 3B). The N protein of HTNV strain 76-118 also co-localized with scFvs in Vero-E6 cell lines with L13F3-ER, L13F3-CYTO, H34-ER, and H34-CYTO antibodies, and L99 viral N protein co-localized only with L13F3 derived intracellular antibodies (Figure 3C).

### 3.4. Inhibition of N protein trafficking reduced HTNV replication

**Figure 5:**
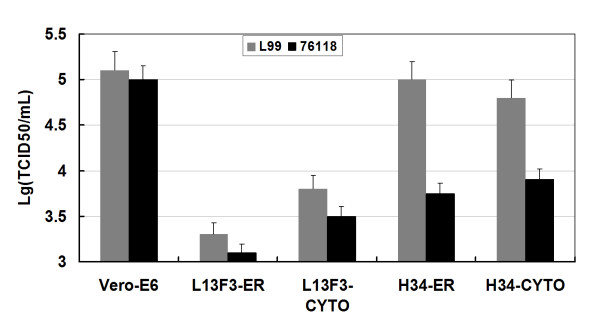
**Titration of viral load in cell culture supernatants by TCID50 assay**. Intracellular antibody-expressing stable cell lines were infected with hantavirus 76-118 and L99 respectively; virus titer at sixth day post infection was determined by the TCID50 assay. Endpoint was determined on day 10, cells fixed on the bottom of the culture plate by 80% (v/v) ice-cold acetone. Viral antigen were detected by horseradish peroxidase conjugated L13F3 monoclonal antibody, virus infection was assessed by absorbance at 450 nm (reference 620 nm). Error bars represent the standard deviation of three independent experiments.

To determine the effect of intracellular antibodies on hantavirus replication, stably transfected L13F3-scFv-ER-E6, H34-scFv-ER-E6, L13F3-scFv-Cyto-E6, and H34-scFv-Cyto-E6 cell lines (1 × 10^6^) were infected with HTNV strain 76-118 and SEOV strain L99 at a MOI of 0.01. Cell culture supernatants were collected each day after the third day postinfection and viral antigen loads were determined by ELISA. Data suggested that HTNV N protein antigen levels in L13F3-scFv-ER-E6 and L13F3-scFv-Cyto-E6 cell lines were lower than that of control cells (Figure 4A). In contrast, N protein antigen levels in stably transfected H34-scFv-ER-E6 and H34-scFv-Cyto-E6 cell lines decreased only when infected with HTNV strain 76-118, but not with SEOV strain L99 (Figure 4B). These data correlate with the antigen-binding specificities of the antibodies. No significant difference of virus replication was observed between stably transfected cell lines and normal non-transfected Vero E6 cells when intracellular antibodies were not specific for the infecting virus. This indicates that intracellular antibodies did not have a negative effect on cell growth, since the SEOV replication rate in H34 antibody-expressing cell lines was similar to that in control Vero E6 cells. HTNV levels in supernatants from the sixth day postinfection were titrated via TCID50 assay. Titers correlated with N protein antigen levels as determined by ELISA (Figure 5). These data indicate that both ER- and cytoplasm-targeted scFv antibodies inhibited HTNV replication. However, HTNV type-specific H34 antibody did not significantly affect replication of SEOV strain L99.

## 4. Discussion

In this study, we demonstrated that N protein of Hantaan and Seoul virus localizes to the ER/*cis*-Golgi in the absence of membrane glycoproteins. Data further suggested that N protein co-localized with intracellular antibodies with the ER retention marker SEKDEL. In addition, analysis of antibody-expressing cell lines, infected with viruses, revealed that N protein trafficking to the ER/*cis*-Golgi is important for viral replication. Blocking of N protein trafficking at both the ER/*cis*-Golgi and in the cytosol leads to inhibition of replication.

The N protein is a 420-430 residue 50-kDa protein, the N-terminal 75 residues of which carry two coiled-coil motifs that facilitate trimerization and nucleocapsid protein trimers that are believed to be HTNV particle assembly intermediates [10,11]. The L13F3 scFv antibody used in this study is known to bind to the N terminal 30 residues of N protein, which comprises part of the first coiled-coil motif [11]. Additionally, H34 scFv has been shown to bind to a conformational epitope.

In hantaviruses, N protein is the first viral protein to accumulate during infection [13,19,20]. Viral RNA segments are complexed with N protein to form individual L, M, and S nucleocapsids [1], which are then packaged into the virion at the bilayered envelope within which are embedded the two viral surface glycoproteins Gn and Gc [21]. The specific interaction between N protein and glycoprotein is thought to trigger budding of virions into the Golgi cisternae and to initiate the virus assembly[4,5]. Although the laboratory evidences were presented recently, the precise mechanism of N protein entry into the ER/*cis*-Golgi apparatus remains unclear. ER-targeted scFvs used for tracking N protein intracellular trafficking were combined with a SEKDEL sequence at the carboxyl terminus. In mammalian cells, SEKDEL receptors are localized primarily to the early Golgi complex at steady state [22,23], but shift in their localization upon binding of SEKDEL-bearing ligands [24]. Therefore, we predicted that the ER-targeted scFvs would follow a route from the ER to the *cis*-Golgi and retrograde transport back to the ER. The co-localization and the proved interaction of N and ER-targeted intracellular antibodies indicate that N protein may entry into the ER/*cis*-Golgi apparatus. During virus infection, N protein or nucleocapsids also co-localized with ER-targeted antibodies. Virus replication was inhibited by the ER-targeted antibodies, which implies that N protein or nucleocapsids presented in membrane cisternae at the ER/*cis*-Golgi apparatus. Previous studies have shown that N protein is membrane-associated [9,14], and that both targeting of N to ERGIC prior to its movement to the Golgi compartment and an intact ERGIC were required for viral replication [14]. In membrane subcellular fractionation experiments, a small proportion of total N protein was detected in membrane-containing fractions [14]. We hypothesize that N protein enters the ER or *cis*-Golgi apparatus, but only with low efficiency. An interaction between ER-targeted scFvs and membrane-associated N protein may act as a "motor" to enhance the entry of N protein into ER/*cis*-Golgi membrane vesicles. Like the function of the interaction of N protein and the cytoplasmic tail of Gn/Gc during virus replication [4,5]. The ER provides a lower-energy environment than the Golgi system [25], which might enhance the entry of cytosol protein or nucleocapsids. The interaction between N and G protein is not the only prerequisite for the entry of N protein or cytosol nucleocapsids into membrane vesicles. Antibodies or other molecules might be alternative driver to direct the N protein to membrane vesicles. Immunoelectron microscopy examination of Uukuniemi virus, a bunyavirus, also demonstrated that the nucleocapsid is associated with membranes that show the characteristic distribution and tubulovesicular morphology of the pre-Golgi intermediate compartment, suggesting that the first site of formation of Uukuniemi virus particles is the pre-Golgi intermediate compartment and that virus budding continues in the Golgi stack [26].

In summary, the data we present in this study suggest that N protein may present in the ER/*cis*-Golgi without the assistance of viral G protein, and that N protein trafficking at these sites plays an important role in HTNV replication.

## Competing interests

The authors declare that they have no competing interests.

## Authors' contributions

LJ, ZQ and WT performed most of the experiments and involved in manuscript preparation. LC was involved in cells culture, virus infection and quantifications. LM participated in the design of the study and editing of the manuscript. LD participated in the design and the analysis of the data and editing of the manuscript. All authors read and approved the final manuscript.

## References

[B1] Obijeski JF Bishop DH Palmer EL Murphy FA Segmented genome and nucleocapsid of La Crosse virus J Virol 1976 20 664 675 994302 10.1128/jvi.20.3.664-675.1976 PMC355044

[B2] Kaukinen P Vaheri A Plyusnin A Hantavirus nucleocapsid protein: a multifunctional molecule with both housekeeping and ambassadorial duties Archives of Virology 2005 V150 1693 1713 10.1007/s00705-005-0555-4 15931462

[B3] Pedersen R Melin L Elliott RM Synthesis, assembly and intracellulat transport of *Bunyaviridae *membrane proteins The Bunyaviridae 1996 New York: Plenum press 159 188

[B4] Wang H Alminaite A Vaheri A Plyusnin A Interaction between hantaviral nucleocapsid protein and the cytoplasmic tail of surface glycoprotein Gn Virus Research 2010 151 205 212 10.1016/j.virusres.2010.05.008 20566401

[B5] Hepojoki J Strandin T Wang H Vapalahti O Vaheri A Lankinen H Cytoplasmic tails of hantavirus glycoproteins interact with the nucleocapsid protein J Gen Virol 2010 91 2341 2350 10.1099/vir.0.021006-0 20444994

[B6] Mir MA Panganiban AT The bunyavirus nucleocapsid protein is an RNA chaperone: Possible roles in viral RNA panhandle formation and genome replication 10.1261/rna.2101906 RNA 2006 12 272 282 10.1261/rna.2101906 16428606 PMC1370907

[B7] Mir MA Panganiban AT Characterization of the RNA Chaperone Activity of Hantavirus Nucleocapsid Protein 10.1128/JVI.00147-06 J Virol 2006 80 6276 6285 10.1128/JVI.00147-06 16775315 PMC1488978

[B8] Xu X Severson W Villegas N Schmaljohn CS Jonsson CB The RNA Binding Domain of the Hantaan Virus N Protein Maps to a Central, Conserved Region 10.1128/JVI.76.7.3301-3308.2002 J Virol 2002 76 3301 3308 10.1128/JVI.76.7.3301-3308.2002 11884555 PMC136036

[B9] Ravkov EV Compans RW Hantavirus Nucleocapsid Protein Is Expressed as a Membrane-Associated Protein in the Perinuclear Region 10.1128/JVI.75.4.1808-1815.2001 J Virol 2001 75 1808 1815 10.1128/JVI.75.4.1808-1815.2001 11160679 PMC114090

[B10] Alfadhli A Love Z Arvidson B Seeds J Willey J Barklis E Hantavirus Nucleocapsid Protein Oligomerization 10.1128/JVI.75.4.2019-2023.2001 J Virol 2001 75 2019 2023 10.1128/JVI.75.4.2019-2023.2001 11160704 PMC115151

[B11] Alfadhli A Steel E Finlay L Bachinger HP Barklis E Hantavirus Nucleocapsid Protein Coiled-Coil Domains 10.1074/jbc.M203395200 J Biol Chem 2002 277 27103 27108 10.1074/jbc.M203395200 12019266

[B12] Kaukinen P Vaheri A Plyusnin A Mapping of the Regions Involved in Homotypic Interactions of Tula Hantavirus N Protein 10.1128/JVI.77.20.10910-10916.2003 J Virol 2003 77 10910 10916 10.1128/JVI.77.20.10910-10916.2003 14512541 PMC225001

[B13] Kariwa H Tanabe H Mizutani T Kon Y Lokugamage K Lokugamage N Iwasa MA Hagiya T Araki K Yoshimatsu K Arikawa J Takashima I Synthesis of Seoul virus RNA and structural proteins in cultured cells Archives of Virology 2003 148 1671 1685 10.1007/s00705-003-0141-6 14505081

[B14] Ramanathan HN Chung D-H Plane SJ Sztul E Chu Y-k Guttieri MC McDowell M Ali G Jonsson CB Dynein-Dependent Transport of the Hantaan Virus Nucleocapsid Protein to the Endoplasmic Reticulum-Golgi Intermediate Compartment 10.1128/JVI.00418-07 J Virol 2007 81 8634 8647 10.1128/JVI.00418-07 17537852 PMC1951367

[B15] Bai X Zhang Y Wang Y Pan L Fack F Bautz E Fine mapping of epitopes of Hantaan virus nucleocapsid protein by peptides scanning Journal of the Fourth Military Medical University 2000 21 36 41

[B16] Liang M Duebel S Li DL Queitschb I Li W Bautz EKF Baculovirus expression cassette vectors for rapid production of complete human IgG from phage display selected antibody fragments Journal of Immunological Methods 2001 247 119 130 10.1016/S0022-1759(00)00322-7 11150543

[B17] Gavrilovskaya IN Brown EJ Ginsberg MH Mackow ER Cellular Entry of Hantaviruses Which Cause Hemorrhagic Fever with Renal Syndrome Is Mediated by beta 3 Integrins J Virol 1999 73 3951 3959 10196290 10.1128/jvi.73.5.3951-3959.1999 PMC104173

[B18] Reed LJ Muench IL A simple method for estimating fifty percent endpoints American Journal of Hygiene 1938 27 493 497

[B19] Hutchinson KL Peters CJ Nichol ST Sin Nombre Virus mRNA Synthesis Virology 1996 224 139 149 10.1006/viro.1996.0515 8862408

[B20] Schmaljohn CS Fields BN, Knipe DM, Howley PM *Bunyaviridae *and their replication Virology 1996 Philadelphia: Lippincott-Raven pub 1447 1471

[B21] Schmaljohn CS Elliott RM Molecular biology of hantaviruses The Bunyaviridae 1996 New York: Plenum Press 63 90

[B22] Lewis MJ Pelham HRB A human homologue of the yeast HDEL receptor 1990 348 162 163 2172835 10.1038/348162a0

[B23] Griffiths G Ericsson M Krijnse-Locker J Nilsson T Goud B Soling H Tang B Wong S Hong W Localization of the Lys, Asp, Glu, Leu tetrapeptide receptor to the Golgi complex and the intermediate compartment in mammalian cells 10.1083/jcb.127.6.1557 J Cell Biol 1994 127 1557 1574 10.1083/jcb.127.6.1557 7798312 PMC2120279

[B24] Lewis MJ Pelham HRB Ligand-induced redistribution of a human KDEL receptor from the Golgi complex to the endoplasmic reticulum Cell 1992 68 353 364 10.1016/0092-8674(92)90476-S 1310258

[B25] Sciaky N Presley J Smith C Zaal KJM Cole N Moreira JE Terasaki M Siggia E Lippincott-Schwartz J Golgi Tubule Traffic and the Effects of Brefeldin A Visualized in Living Cells 10.1083/jcb.139.5.1137 J Cell Biol 1997 139 1137 1155 10.1083/jcb.139.5.1137 9382862 PMC2140213

[B26] Jantti J Hilden P Ronka H Makiranta V Keranen S Kuismanen E Immunocytochemical analysis of Uukuniemi virus budding compartments: role of the intermediate compartment and the Golgi stack in virus maturation J Virol 1997 71 1162 1172 8995638 10.1128/jvi.71.2.1162-1172.1997 PMC191169

